# Association Between Acupuncture’s Neuroprotective Effects and Integrin Alpha 7

**DOI:** 10.3390/medicina62040670

**Published:** 2026-04-01

**Authors:** Sangeun Han, Sabina Lim, Sujung Yeo

**Affiliations:** 1 Department of Korean Medicine Data Division, Korea Institute of Oriental Medicine, Daejeon 34054, Republic of Korea; sadgc0303@kiom.re.kr; 2 Department of Korean Convergence Medical Science, University of Science & Technology (UST), Daejeon 34113, Republic of Korea; 3 Department of Meridian and Acupoint, College of Korean Medicine, Kyung Hee University, Seoul 02453, Republic of Korea; 4 Research Institute of Korean Medicine, Sang Ji University, Wonju 26339, Republic of Korea

**Keywords:** acupuncture, LR3, GB34, Parkinson’s disease, alpha-synuclein, ITGA7, substantia nigra, MPTP

## Abstract

*Background and Objectives*: Parkinson’s disease (PD) entails the progressive degeneration of dopaminergic neurons in the substantia nigra (SN), accompanied by α-synuclein (α-syn)-enriched Lewy bodies. ITGA7 mediates cell–extracellular matrix adhesion and modulates apoptosis, though its involvement in PD pathogenesis warrants further investigation. Although acupuncture demonstrates neuroprotective effects in PD models, its precise molecular mechanisms remain incompletely understood; therefore, in this study, we explored the relationship between ITGA7 and α-synuclein expression in an MPTP-induced PD mouse model to determine the association between LR3/GB34 acupuncture-induced changes in α-synuclein levels and ITGA7 modulation. *Materials and Methods*: In the in vivo model, MPTP-induced PD mice underwent immunohistochemistry, immunofluorescence, and Western blotting to assess ITGA7, α-synuclein, and TH levels in the SN and striatal tissues following LR3/GB34 acupuncture. In parallel, for the in vitro mechanistic study, SH-SY5Y neuroblastoma cells treated with MPP^+^ and transfected with ITGA7-siRNA were utilized to examine the involvement of apoptosis-related signaling pathways. *Results*: In the in vivo model, MPTP administration downregulated ITGA7 and upregulated α-synuclein in SN tissues. Similarly, in vitro exposure of SH-SY5Y cells to MPP^+^ yielded comparable results, revealing an inverse correlation between ITGA7 and α-synuclein. LR3/GB34 acupuncture treatment in the mouse model significantly increased ITGA7 and TH expression while reducing α-synuclein accumulation. To further understand the specific role of ITGA7 observed in these animal findings, we silenced ITGA7 in the MPP^+^-treated cellular model. ITGA7 silencing exacerbated the neurotoxic effects, leading to further TH downregulation, α-synuclein upregulation, Bcl-2 reduction, and Bax/Bcl-2 ratio elevation. *Conclusions*: Collectively, the histological preservation of dopaminergic neurons following LR3/GB34 acupuncture in the PD mouse model appears to be linked to ITGA7 upregulation. Furthermore, our in vitro findings implicate ITGA7 in the regulation of apoptosis-related signaling cascades, supporting its potential role in mitigating α-synuclein pathology.

## 1. Introduction

Parkinson’s disease (PD) is a slow progressive disorder of the nervous system in which damage to dopaminergic neurons in the substantia nigra (SN) gives rise to characteristic motor symptoms. Patients typically present with reduced movement speed, a resting tremor, and muscle stiffness [1]. A key neuropathological hallmark of PD is the presence of aberrant alpha-synuclein (α-syn) aggregates within Lewy bodies [2]. α-Syn is a presynaptic protein implicated in dopaminergic neuronal death [3], known to propagate extensively throughout multiple brain regions [4]. Its increased expression has been consistently observed in both PD patients and animal models [5]. In addition to classic mechanisms such as neuroinflammation and oxidative stress, metabolic disturbances have recently been recognized as critical factors influencing PD pathogenesis. Notably, hypoglycemic episodes and glycemic variability have been highlighted for their significant impact on the progression and exacerbation of Parkinsonian syndromes [6].

Recent evidence suggests that decreased expression of integrin alpha 7 (ITGA7) may contribute to elevated α-syn levels in the substantia nigra (SN) of long-term MPTP-treated PD mice, and reduced ITGA7 expression has been associated with apoptosis of dopaminergic neurons [7]. Furthermore, ITGA7 deficiency in muscle tissue leads to α-syn accumulation, resulting in impaired muscle cell production and delayed muscle differentiation recovery [8]. Because aberrant α-syn accumulation is a shared pathological hallmark across a spectrum of neurodegenerative disorders, the significance of ITGA7 expression likely extends beyond PD to other synucleinopathies, such as multiple system atrophy (MSA) and dementia with Lewy bodies (DLB) [9,10]. ITGA7 is encoded by the ITGA7 gene [11] and is expressed in multiple tissues, including the brain, skeletal muscle, and heart [12]. Functionally, it plays a critical role in facilitating cell-to-cell adhesion and cell–matrix binding, thereby modulating cellular proliferation, migration, and programmed cell death [13].

Acupuncture has attracted increasing attention as a complementary therapeutic approach for various conditions, including fibromyalgia, myofascial pain, and PD [14]. Acupuncture at Taichong (LR3) has activated brain activity in patients with vascular dementia [15], while stimulation at Yanglingquan (GB34) has improved motor function in stroke patients and activated motor-related brain regions [16,17]. It is also known to modulate the gene expression of neuropeptides [18]. In MPTP-induced PD mouse models, acupuncture treatment at LR3 and GB34 has been reported to upregulate gene expression in the thalamus and to inhibit the reduction in tyrosine hydroxylase (TH) expression in thalamic regions, suggesting a neuroprotective effect against MPTP-induced damage [19]. In addition, acupuncture suppresses MPTP-induced dopaminergic neuronal cell death [20] and inhibits pathological α-syn upregulation [21].

From these observations, we hypothesized that the role of ITGA7 in apoptosis-related signaling may be associated with the neuroprotective effects of acupuncture through gene expression regulation; however, the relationship between acupuncture-induced neuroprotection and the involvement of ITGA7 in apoptosis-related pathways has not yet been fully elucidated.

Therefore, in this study, we aimed to identify a novel mechanism underlying the effects of LR3 and GB34 acupuncture in Parkinson’s disease by investigating ITGA7 and α-syn levels in the SN of an MPTP-induced PD mouse model after acupuncture.

## 2. Materials and Methods

### 2.1. MPTP-Induced PD Mouse Model

Following a 1-week acclimation period, a total of 24 four-week-old male inbred C57BL/6 mice (20–22 g; DBL, Seoul, Republic of Korea) were housed in standard plastic cages (6 mice per cage) in a controlled environment at 24 °C with a 12 h light/dark cycle. They were provided with free access to a standard laboratory diet and water. The mice were randomly assigned to four experimental groups (n = 6 per group) using a computer-generated random number sequence to ensure no significant baseline differences: control (C), MPTP (M), acupuncture (A), and non-acupoint (NA). Mice in the C group received intraperitoneal injections of 0.9% saline (100 μL), while the MPTP, Acu, and Non-Acu groups received daily intraperitoneal MPTP-HCl injections (20 mg/kg free base; Sigma, St. Louis, MO, USA) over four consecutive weeks to establish Parkinson’s disease pathology. At 24 h after the final MPTP injection, corresponding to 48 h after the last acupuncture session, the mice were euthanized for tissue collection. All experimental procedures were conducted in accordance with protocols approved by the Sangji University Institutional Animal Care and Use Committee (IACUC No. 2021-9).

### 2.2. LR3/GB34 Acupuncture Application

Treatment began 2 h following the initial MPTP administration in both the acupuncture (A) and non-acupoint (NA) groups. In the former, acupuncture needles were bilaterally inserted at Taichong (LR3) to a depth of 1 mm and at Yanglingquan (GB34) to 3 mm. In the NA group, which served as a sham control to account for handling and needle insertion stress, needles were bilaterally inserted to a depth of 3 mm at non-acupoints in the gluteal muscle (approximately 5 mm lateral to the midline), strictly avoiding any known meridian lines. Acupuncture stimulation was manually applied a total of 14 times at 48 h intervals, with the retention time and manipulation matching that of the A group exactly to ensure consistent stimulation intensity.

### 2.3. SH-SY5Y Cell Lines and Culture Conditions

SH-SY5Y human neuroblastoma cells were maintained in minimum essential medium (MEM; Welgen, Namcheon-myeon, Republic of Korea) supplemented with 10% fetal bovine serum (FBS; Lonza, Walkersville, MD, USA), 100 U/mL penicillin, and 100 μg/mL streptomycin. Cultures were grown at 37 °C in a humidified 5% CO_2_ incubator.

### 2.4. ITGA7 siRNA Transfection Protocol

Stealth siRNA targeting ITGA7 (5′-GAC AUG CAC UAC CUC GUC U-3′) and scrambled negative control siRNA (5′-UUC UCC GAA CGU GUC ACG UTT-3′) were obtained from Bioneer Inc. (Daejeon, Republic of Korea). Prior to transfection, SH-SY5Y cells were cultured in Opti-MEM medium (Gibco, Amarillo, TX, USA), and then transfection was performed using Promega transfection reagent (Madison, WI, USA) at a 3.5:1 reagent-to-siRNA ratio, followed by 24 h incubation.

### 2.5. MPP^+^ Treatment

In vitro neurotoxicity was induced by treating SH-SY5Y cells with 500 μM MPP^+^ iodide (Sigma, St. Louis, MO, USA) for 18 h.

### 2.6. Western Blot Analysis

Tissues from the substantia nigra (SN), striatum (ST), and SH-SY5Y cell pellets were lysed in RIPA buffer on ice for 30 min. Lysates underwent centrifugation at 12,000 rpm and 4 °C for 20 min, followed by protein quantification via the bicinchoninic acid (BCA) assay. Equal protein amounts were resolved by SDS-PAGE on 4–15% Tris-Bis gels and electroblotted onto PVDF membranes. Membranes were blocked with 3% BSA at 37 °C for 90 min, and then incubated overnight at 4 °C with primary antibodies against ITGA7 (1:1000; Santa Cruz sc-515716), TH (1:2000; Santa Cruz sc-25269), Bcl-2 (1:1000; Santa Cruz sc-25269), Bax (1:1000; Abcam ab32503), α-synuclein (1:500; Novus NBP2-15365), and β-actin (1:5000; Santa Cruz sc-47778). After undergoing TBST washes, membranes were incubated with HRP-conjugated secondary antibodies (1:5000; Santa Cruz) for 1 h at room temperature. Protein bands were detected using chemiluminescence and quantified with ImageJ software (version 1.54d).

### 2.7. Immunohistochemistry

Brain tissues were immersed in 4% paraformaldehyde in 0.05 M sodium phosphate buffer at 4 °C for 24 h, and then cryoprotected in sucrose solution at 4 °C for 48 h, after which forty-micrometer-thick sections were obtained using a cryomicrotome. Coronal brain sections encompassing the striatum (Bregma +1.10 to +0.14 mm) and substantia nigra (Bregma −2.92 to −3.52 mm) were selected according to the mouse brain atlas. Sections were incubated overnight at 4 °C with primary antibodies targeting ITGA7 (1:100), α-synuclein (1:500), or TH (1:2000). Following incubation with biotinylated anti-mouse IgG, sections were treated with an avidin–biotin peroxidase complex and developed using the diaminobenzidine (DAB) chromogen. Representative images from each group (n = 3 mice per group) were utilized to qualitatively assess protein distribution, while quantitative validation of protein expression was strictly conducted via independent Western blot analyses.

### 2.8. Immunofluorescence Analysis

Tissue sections were incubated with primary antibodies followed by biotinylated anti-mouse IgG and fluorescein avidin DCS (Vector Laboratories, Burlington, ON, Canada) to detect ITGA7 (1:100) and α-synuclein (1:200). Sections then received the avidin/biotin blocking kit and mouse-on-mouse (M.O.M.) IgG blocking reagent (Vector Laboratories) prior to overnight incubation at 4 °C with anti-α-synuclein or anti-ITGA7 IgG. Subsequently, sections were treated with biotinylated anti-mouse IgG and rhodamine avidin D, followed by imaging on a Nikon X-Cite Series 120Q fluorescence microscope (Nikon, Tokyo, Japan) using consistent exposure parameters across all groups.

### 2.9. Statistical Analysis

Data were analyzed using GraphPad Prism software (version 10.0, GraphPad Software, Boston, MA, USA). For comparisons between two groups, an unpaired Student *t*-test was used, while among three or more groups, a one-way analysis of variance (ANOVA) was performed. To correct for multiple comparisons, Tukey’s post hoc test was applied for all in vivo experiments (comparing all experimental groups), whereas Dunnett’s post hoc test was utilized for in vitro dose–response experiments (comparing treatment groups to the negative control). Prior to ANOVA, data distribution and variance were checked to ensure that they met the assumptions for parametric tests. The results of these experiments are expressed as mean ± standard error of the mean (SEM), where “n” denotes the number of biological replicates: individual animals (in vivo) or independent cell culture experiments (in vitro). No data points were excluded from the analysis and statistical significance was set at *p* < 0.05, exact *p*-values are reported where applicable.

## 3. Results

### 3.1. TH Expression Reduction in Chronic MPTP-Induced Parkinsonian Mice

**Figure 1:**
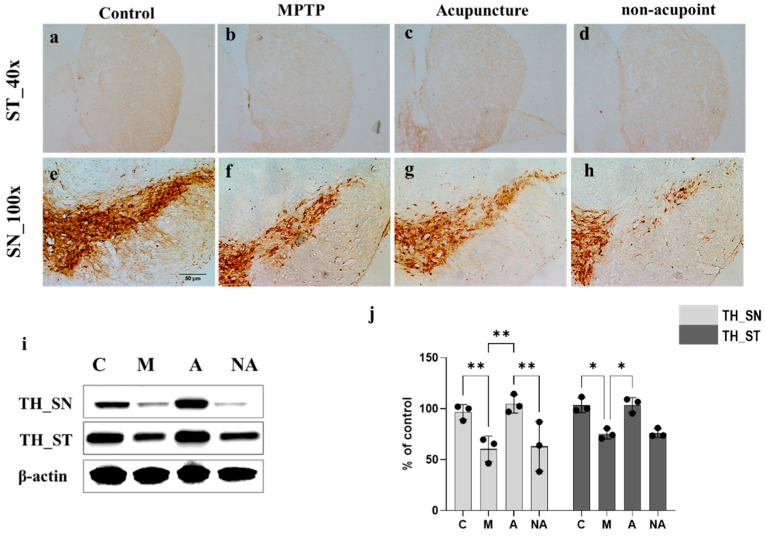
Tyrosine hydroxylase (TH) protein levels in the MPTP-induced Parkinson’s disease (PD) in vivo mouse model. To assess dopaminergic neuronal survival, immunohistochemical staining for TH was performed in the striatum (ST; (**a**–**d**)) and substantia nigra (SN; (**e**–**h**)) across four experimental groups: control (C), MPTP-treated (M), MPTP + LR3/GB34 acupuncture (A), and MPTP + non-acupoint (NA) groups. Prominent TH reduction was evident in the ST and SN of both the M and NA groups, and Western blot quantification (**i**,**j**) confirmed significant TH downregulation in the M group relative to the C group (*p* = 0.0063 for SN; *p* = 0.0362 for ST), though acupuncture treatment significantly attenuated this reduction (*p* = 0.0011 for SN; *p* = 0.0337 for ST vs. M). Furthermore, TH levels in the A group were significantly higher than those in the NA group (*p* = 0.0019 for SN; *p* = 0.0398 for ST). Data represent mean ± SEM (n = 3) and statistical significance was analyzed by one-way ANOVA followed by Tukey’s multiple comparisons test. * *p* < 0.05, ** *p* < 0.01.

Saline or MPTP was administered daily via intraperitoneal injection to the control, MPTP, acupuncture (A), and non-acupoint (NA) groups, while acupuncture was applied 2 h post-MPTP injection at designated acupoints for the A and NA groups. Following 4 weeks of treatment, tyrosine hydroxylase (TH) levels in the substantia nigra (SN) and striatum (ST) were assessed to validate the chronic MPTP-PD mouse model. Immunohistochemistry demonstrated marked TH reduction in both the SN and ST of the M group (Figure 1b,f) relative to controls (Figure 1a,e). Notably, acupuncture treatment significantly mitigated the reduction in TH expression (Figure 1c,g) compared to the M group (*p* = 0.0011 for SN; *p* = 0.0337 for ST), a finding corroborated by Western blotting showing significant TH downregulation in the M group versus the C group (*p* = 0.0063 for SN; *p* = 0.0362 for ST). Importantly, significant recovery was observed specifically in the A group compared to both the M and NA groups (*p* = 0.0019 for SN and *p* = 0.0398 for ST in A vs. NA; n = 3 per group; Figure 1i,j).

### 3.2. Increased ITGA7 Expression Following Acupuncture Treatment in MPTP-Induced PD Mouse Model

**Figure 2:**
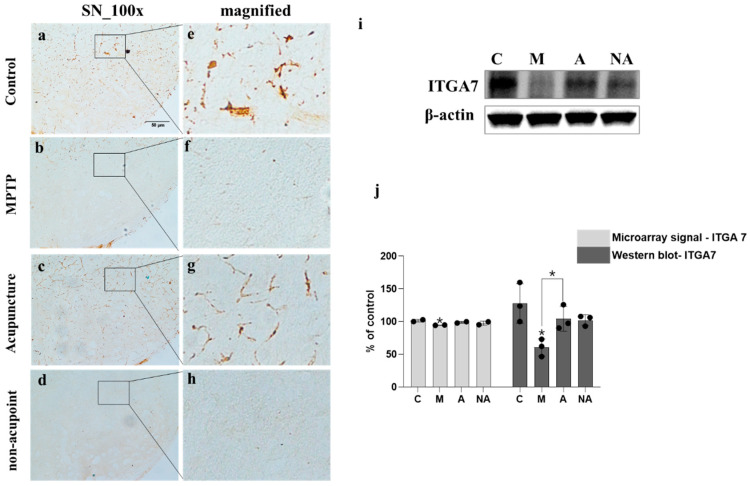
Modulation of integrin alpha-7 (ITGA7) expression by acupuncture in the MPTP-induced PD mouse model. To investigate the in vivo association between acupuncture and ITGA7, immunohistochemical detection of ITGA7 was conducted in the substantia nigra (SN; (**a**–**d**)) across the control (C), MPTP (M), MPTP + acupuncture (A), and MPTP + non-acupoint (NA) groups, with higher-magnification views of corresponding SN regions shown in (**e**–**h**). ITGA7 immunoreactivity was diminished in M and NA groups and elevated in C and A groups. Western blot data (**i**,**j**) validated these observations, confirming significant reduction in the M group (*p* = 0.0111 vs. C) and significant restoration in the A group (*p* = 0.0293 vs. M). Microarray results also supported this expression pattern (*p* < 0.05, n = 2). Values represent mean ± SEM, and statistical significance for Western blot data was analyzed by one-way ANOVA followed by Tukey’s multiple comparisons test, while microarray data were compared using Student’s *t*-test. * *p* < 0.05.

Immunohistochemistry revealed marked ITGA7 downregulation in the SN of the MPTP (M) (Figure 2b,f) relative to the control group (C) (Figure 2a,e). The acupuncture group (A) exhibited a significant recovery in ITGA7 levels (Figure 2c,g) compared to the MPTP group, whereas the non-acupoint group (NA) showed persistent ITGA7 reduction (Figure 2d,h). Western blotting corroborated these observations, demonstrating a significant ITGA7 decrease in the M versus the C group (*p* = 0.0111) and a significant recovery in the A group (*p* = 0.0293 vs. M; n = 3; Figure 2i,j). Furthermore, microarray analysis, used for initial gene expression screening, also confirmed a significant modulation in ITGA7 following treatment (*p* < 0.05, n = 2; analyzed by Student’s *t*-test).

### 3.3. Decreased α-Syn Expression Following Acupuncture Treatment in MPTP-Induced PD Mouse Model

**Figure 3:**
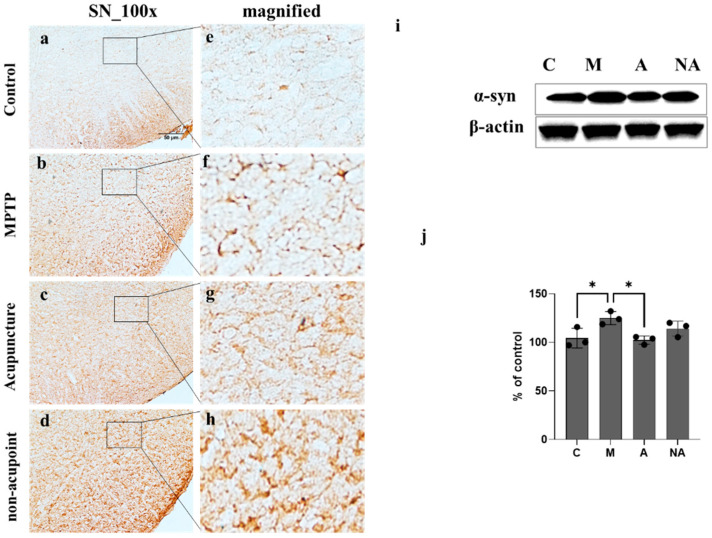
Modulation of α-synuclein (α-syn) accumulation by acupuncture in the MPTP-induced PD mouse model. To evaluate pathological protein aggregation in vivo, immunohistochemical localization of α-syn was performed in the substantia nigra (SN; (**a**–**d**)) of the control (C), MPTP (M), MPTP + acupuncture (A), and MPTP + non-acupoint (NA) groups. High-magnification images of respective SN areas are depicted in (**e**–**h**). α-Syn immunoreactivity was prominently elevated in the M group, contrasting with reduced levels in the C and A groups. Western blot results (**i**,**j**) verified significant α-syn upregulation in the M versus the C group (*p* = 0.0397) and significant reduction in the A group compared to the M group (*p* = 0.0259). Data are shown as mean ± SEM (n = 3). Statistical significance was analyzed by one-way ANOVA followed by Tukey’s multiple comparisons test. * *p* < 0.05.

Immunohistochemistry revealed pronounced α-synuclein (α-syn) upregulation in the SN of mice in the MPTP group (Figure 3b,f) relative to controls (Figure 3a,e), with acupuncture treatment significantly attenuating α-syn levels (Figure 3c,g) compared to the M group. Western blot quantification confirmed significant α-syn elevation in the M versus the C group (*p* = 0.0397), with significant suppression observed in the A group (*p* = 0.0259 vs. M; n = 3; Figure 3i,j).

### 3.4. Immunoblot Analysis of TH/ITGA7/Bcl-2 Expression in ITGA7-siRNA-Transfected SH-SY5Y Cells

**Figure 4:**
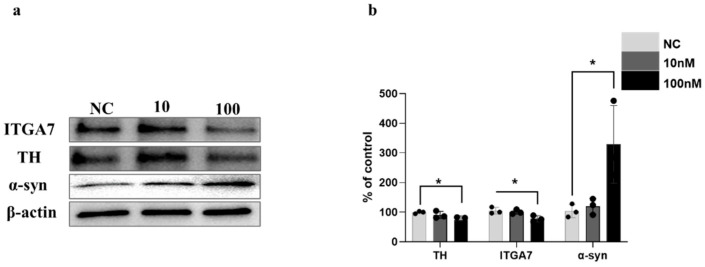
Effect of ITGA7 silencing on α-synuclein (α-syn) and tyrosine hydroxylase (TH) expression in the in vitro SH-SY5Y cellular model. To establish the mechanistic link between ITGA7 and PD-related markers, SH-SY5Y neuroblastoma cells were transfected with ITGA7-specific small interfering RNA (siRNA). (**a**,**b**) Western blot images (**a**) and quantitative analysis (**b**) demonstrate reduced TH and ITGA7 levels alongside elevated α-synuclein in SH-SY5Y cells following ITGA7 siRNA transfection. NC: negative control siRNA (100 nM, 24 h); 10: ITGA7 siRNA (10 nM, 24 h); 100: ITGA7 siRNA (100 nM, 24 h). Data are shown as mean ± SEM (n = 3) and statistical significance was analyzed by one-way ANOVA followed by Dunnett’s multiple comparisons test (* *p* < 0.05 vs. NC). Exact *p*-values for the 100 nM treatment group compared to the NC group are *p* = 0.0487 (TH), *p* = 0.0263 (ITGA7), and *p* = 0.0221 (α-syn) * *p* < 0.05.

**Figure 5:**
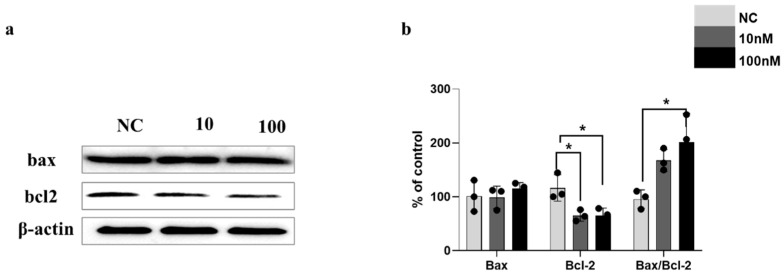
Knockdown of ITGA7 modulates apoptosis-related signaling pathways in SH-SY5Y cells. To explore the downstream survival mechanisms in vitro, apoptosis-related proteins were analyzed following ITGA7 silencing. (**a**,**b**) Western blot images (**a**) and quantitative analysis (**b**) reveal downregulated anti-apoptotic Bcl-2 and an increased Bax/Bcl-2 ratio in SH-SY5Y cells after ITGA7 siRNA treatment. NC: negative control siRNA (100 nM, 24 h); 10: ITGA7 siRNA (10 nM, 24 h); 100: ITGA7 siRNA (100 nM, 24 h). Data represent mean ± SEM (n = 3) and statistical significance was analyzed by one-way ANOVA followed by Dunnett’s multiple comparisons test (* *p* < 0.05 vs. NC). Exact *p*-values for 100 nM treatment compared to the NC group are *p* = 0.6412 (Bax), *p* = 0.0196 (Bcl-2), and *p* = 0.0158 (Bax/Bcl-2 ratio). * *p* < 0.05.

ITGA7 siRNA transfection markedly reduced ITGA7 and TH protein levels in SH-SY5Y cells (*p* = 0.0263 for ITGA7 and *p* = 0.0487 for TH at 100 nM vs. NC; n = 3). Concurrently, α-syn expression was substantially upregulated post-knockdown (*p* = 0.0221 at 100 nM vs. NC; n = 3; Figure 4a,b) and ITGA7 silencing also suppressed Bcl-2 expression (*p* = 0.0196 at 100 nM vs. NC), while Bax levels remained unchanged (*p* = 0.6412 at 100 nM vs. NC). Nevertheless, the Bax/Bcl-2 ratio significantly increased following ITGA7 siRNA treatment (*p* = 0.0158 at 100 nM vs. NC; n = 3; Figure 5a).

### 3.5. Immunofluorescent Examination of ITGA7 and α-Synuclein in SH-SY5Y Cells

**Figure 6:**
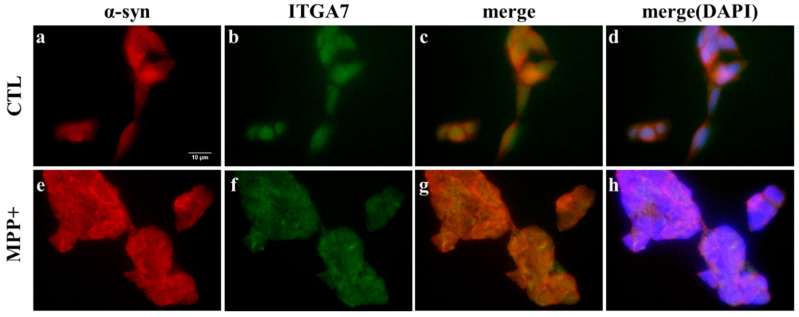
Co-localization of ITGA7 and α-synuclein (α-syn) in the in vitro MPP^+^-induced neurotoxicity model. To visualize the spatial relationship between ITGA7 and α-syn under neurotoxic conditions, SH-SY5Y cells were treated with MPP^+^. Fluorescence micrographs depict ITGA7 (green; (**b**,**f**)) and α-synuclein (red; (**a**,**e**)) labeling via fluorescein avidin and rhodamine avidin, respectively. Merged fluorescence is shown in (**c**,**g**). Compared to untreated controls (**c**), MPP^+^ treatment visibly reduced ITGA7 while enhancing α-syn signals (**g**), with distinct colocalization evident in untreated cells. DAPI-stained nuclei appear in (**d**,**h**).

Immunofluorescence staining confirmed ITGA7/α-Syn colocalization in SH-SY5Y cells, with ITGA7 fluorescence intensity being substantially higher in untreated controls compared to MPP^+^-exposed cells (Figure 6b,f). Conversely, the α-Syn signal was prominently elevated in the MPP^+^ group versus controls (Figure 6a,e). Through merged images, we highlight predominant ITGA7 expression in controls and α-synuclein dominance in MPP^+^-treated cells (Figure 6c,g), with DAPI nuclear counterstaining visible in Figure 6d,h.

## 4. Discussion

To identify a novel mechanism underlying the effects of LR3 and GB34 acupuncture on Parkinson’s disease, in this study, we investigated ITGA7 and α-syn protein levels within the substantia nigra (SN) regions of MPTP-PD mice using immunohistochemical, immunofluorescence, and Western blot analyses. Acupuncture is widely recognized as a complementary therapy for various diseases, including fibromyalgia, myofascial pain, and Parkinson’s disease [14], and is known to alter neuropeptide transcription [18]. In particular, acupuncture has been shown to suppress α-synuclein accumulation within nigra areas of MPTP-PD rodents while enhancing dopaminergic neuron viability [20,21].

Consistent with previous findings, tyrosine hydroxylase (TH) levels were markedly reduced in both the striatum (ST) and SN territories of MPTP-treated compared with control mice. However, LR3/GB34 acupuncture markedly counteracted MPTP-mediated TH depletion in ST and SN regions (Figure 1), suggesting its potential role in attenuating MPTP-driven dopaminergic injury. MPTP exposure significantly downregulated ITGA7 expression levels in the SN, which was effectively mitigated following acupuncture treatment (Figure 2). ITGA7 encodes an extracellular matrix (ECM)-adhering integrin—a binding protein that functions as part of the integrin superfamily of transmembrane glycoproteins—thereby facilitating critical cell–ECM interactions essential for proper apoptosis regulation [22]. Previous studies have demonstrated that diminished ITGA7 expression in neural and muscular tissues is closely associated with α-synuclein overexpression, particularly implicating the ITGA7-α-synuclein signaling pathway as being strongly involved in Parkinson’s disease (PD) progression [7,8]. Consistent with these findings, our results showed that acupuncture treatment was associated with the attenuation of MPTP-induced downregulation of ITGA7 protein levels in the SN region, potentially contributing to the restoration of ITGA7 protein expression. In parallel, α-syn expression significantly increased following MPTP treatment, whereas this increase was markedly attenuated following acupuncture (Figure 3). α-Syn is a small natively unfolded protein predominantly enriched in presynaptic nerve terminals, and its increased expression is considered a critical step in the pathogenesis of Parkinson’s disease [23,24]. Although the physiological role of α-syn has not been fully elucidated, its abnormal accumulation is a hallmark of α-synucleinopathies, including Parkinson’s disease [25]. These results further support the potential role of acupuncture in attenuating the MPTP-induced pathological accumulation of α-syn in the SN. To further investigate the relationship between ITGA7 loss and increased α-syn expression, ITGA7 was knocked down in SH-SY5Y cells using ITGA7-specific siRNA. Reduction in ITGA7 expression in SH-SY5Y cells led to decreased TH expression and increased α-syn expression (Figure 4). Moreover, ITGA7 knockdown resulted in decreased anti-apoptotic protein Bcl-2 expression and a significant elevation in the Bax/Bcl-2 ratio (Figure 5). Elevated Bax levels promote apoptosis by facilitating cytochrome c release from the mitochondrial intermembrane space into the cytosol, whereas Bcl-2 inhibits this process. The precise mechanism by which ITGA7 regulates Bcl-2 warrants further analysis: Integrins classically mediate cell survival by activating downstream effectors, such as focal adhesion kinase (FAK) and the PI3K/Akt pathway, which in turn promote the transcription and stabilization of anti-apoptotic Bcl-2 family proteins. Therefore, the knockdown of ITGA7 likely disrupts these essential cell–matrix survival signals, resulting in decreased Bcl-2 expression [26,27], which shifts the cellular balance, driving a significant elevation in the Bax/Bcl-2 ratio and initiating apoptosis-related cascades. These results suggest that ITGA7 is closely associated with α-syn accumulation and apoptosis-related signaling in SH-SY5Y cells. Furthermore, given this close regulatory relationship with α-synuclein, the significance of ITGA7 expression may extend beyond PD; modulating ITGA7 could hold broad translational potential for other synucleinopathies, such as multiple system atrophy (MSA) and dementia with Lewy bodies (DLB), warranting further investigation into its pan-synucleinopathy neuroprotective effects [9,10].

Immunofluorescence analysis revealed that ITGA7 and α-syn predominantly localized to the cell periphery in SH-SY5Y cells, with MPP^+^ treatment causing increased α-syn and decreased ITGA7 expression relative to the control group (Figure 6). Notably, ITGA7 expression was inversely correlated with α-syn expression, indicating that reduced ITGA7 levels are associated with enhanced α-syn accumulation.

While our findings indicate a strong association between acupuncture and ITGA7 upregulation, the exact upstream mechanisms bridging physical needle stimulation and ITGA7 expression require further exploration. It is plausible that localized mechanical stimulation at the acupoint induces mechanotransduction, which subsequently modulates integrin pathways and neuromodulatory networks [28]. Importantly, the singular impact of acupuncture on ITGA7 could be jeopardized by co-existing systemic factors; for instance, severe metabolic disorders (such as glycemic variability), chronic stress, or concurrent pharmacological treatments may alter the baseline neuroinflammatory state, potentially dampening the mechanotransduction efficiency and the subsequent neuroprotective signaling resulting from acupuncture [6].

Several limitations of this study should be critically acknowledged. First, we utilized an MPTP-induced mouse model, which primarily mimics acute dopaminergic toxicity and may not fully replicate the slow, progressive pathogenesis of human PD, including the complex metabolic and environmental variables seen in patients. Second, although we established the mechanistic role of ITGA7 using siRNA-mediated knockdown in vitro, we did not employ ITGA7-knockout or transgenic mouse models to validate these findings in vivo. Future studies utilizing such genetic animal models are required to definitively elucidate the systemic role of ITGA7 in acupuncture-mediated neuroprotection. Lastly, while we observed an association between ITGA7 upregulation and α-synuclein reduction, the precise upstream pathways linking mechanical acupuncture stimulation to ITGA7 transcriptional activation remain unidentified. Furthermore, due to the experiments and data analyses being primarily conducted by a single investigator, rigorous blinding during tissue processing and histological quantification was not feasible, which may have introduced a potential risk of investigator bias. Future studies employing a multi-investigator, double-blinded approach are warranted to robustly validate these findings.

Taken together, these findings suggest that the involvement of ITGA7 in apoptosis-related signaling is closely linked to α-syn accumulation and dopaminergic neuronal loss, and that the neuroprotective effects following acupuncture stimulation at LR3 and GB34 may be closely associated with the modulation of ITGA7 expression in the SN and the subsequent attenuation of α-syn accumulation.

## 5. Conclusions

In this study, we demonstrated an association between the histological preservation of dopaminergic neurons following acupuncture and ITGA7 expression. In the SN region of Parkinson’s disease mice, ITGA7 expression was decreased following MPTP treatment but showed recovery following acupuncture at LR3 and GB34; conversely, the MPTP-induced increase in α-syn expression was significantly mitigated following acupuncture at the same acupoints. Furthermore, knockdown of ITGA7 using siRNA resulted in increased α-syn expression in SH-SY5Y and an altered Bax/Bcl-2 ratio in SH-SY5Y neuroblastoma cells, suggesting that ITGA7 downregulation likely contributes to α-syn accumulation and apoptosis-related signaling pathway activation in the SN. Taken together, the present results suggest that the attenuation of dopaminergic injury associated with acupuncture may be mediated, at least in part, by the modulation of ITGA7 expression and the associated reduction in α-syn expression.

## Data Availability

All data analyzed in this study are included in this published article.
